# Abdominal abscess complicating peritonitis in a peritoneal dialysis patient

**DOI:** 10.1590/2175-8239-JBN-2021-0131

**Published:** 2021-12-06

**Authors:** Cátia Raquel Figueiredo, Hernâni Gonçalves, Francisco Ferrer

**Affiliations:** 1 Centro Hospitalar do Médio Tejo, EPE, Serviço de Nefrologia, Torres Novas, Santarém, Portugal.

Dear Editor, 

Peritonitis is the most serious infectious complication of peritoneal dialysis (PD), causing high morbidity and mortality, especially when complicated with an intra-abdominal abscess. The prognosis of PD-related peritonitis is closely associated with the patient's functional status and causative pathogens^1^. Nearly 0.7% of peritonitis complicates with intra-abdominal abscesses that can be attributed to concomitant or previous peritonitis episodes^2^. In a cohort study, the overall in-hospital mortality of liver abscesses in patients on dialysis was 10.1%^3^.

**Figure 1:**
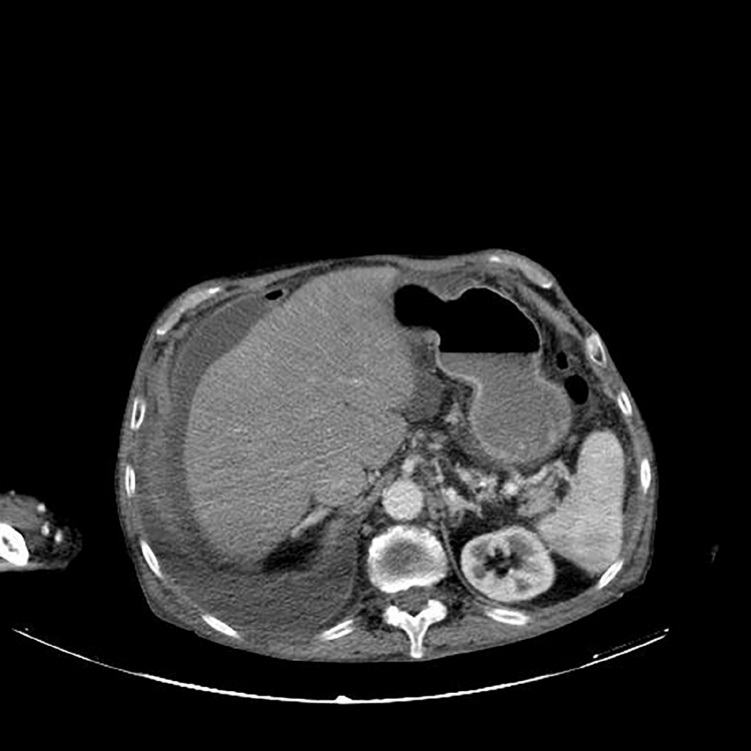
A capsulated abdominal collection, suggestive of an abscess, in the liver.

We present a case of a 77-year-old man with severe heart failure and cardiorenal syndrome who was in assisted peritoneal dialysis for ten months to control refractory hypervolemia. While maintaining PD, he presented two infectious episodes to methicillin-resistant *Staphylococcus aureus* (MRSA): an exit-site infection and a peritonitis, 5 months later, both treated with intra-peritoneal vancomycin 1 g for 21 days. After two months, the patient was admitted to the nephrology department because of abdominal pain in the right hypochondrium. The peritoneal fluid was turbid and the cytological examination revealed a diagnosis of peritonitis (leukocytes: 1700/ µL and 80% neutrophils). Double intraperitoneal antibiotherapy with vancomycin 1g and ceftazidime 1g was initiated immediately. The effluent microbiological testing was positive for MRSA, and he maintained the antibiotherapy with vancomycin only, according to the antibiogram (minimum inhibitory concentration=1µg/mL). The PD catheter was immediately removed, but the patient evolved with hypotension (mean arterial pressure <70 mmHg), altered mental status, and liver dysfunction (total bilirubin: 14.8 mg/dL; albumin: 1.7 g/dL, and platelets 110.000/µL) which gave a Sequential Organ Failure Assessment (SOFA) score of 10. Given the multiple comorbidities and daily living dependency previous to hospitalization, the patient did not meet the criteria for intensive care. A Computerized Tomography (CT) scan was performed on the 8^th^ day, which revealed a capsulated abdominal collection, suggestive of an abscess (Figure 1), from the right lobe of the liver to the supravesical region. Considering the high risk of bleeding (International Normalized Ratio of 1.9), the patient was rejected for percutaneous drainage and also for surgery because of his severe heart failure. He died 10 days after under comfort measures.

This case reports a patient with an abdominal abscess complicating MRSA peritonitis. In a published study that included all adult Australian PD patients, 22% of peritonitis episodes were caused by MRSA^4^. This is a serious complication of PD, associated with poor outcomes, such as hospitalizations, high drop-out rates and death. It can cause intra-abdominal complications and its role in the development of peritoneal abscesses has already been demontrated in a basic research^5^.

In this case, there was no improvement after intra-peritoneal antibiotherapy and catheter removal, which lead to the suspicion of intra-peritoneal complication. Unfortunately, the patient's comorbidities and unfavorable evolution during hospitalization already predicted the worst outcome. It is doubtful whether a more aggressive approach after the first episode of peritonitis could have prevented abscess development. However, we want to emphasize the importance of being aware of this complication, which is rare but should be suspected if symptoms do not resolve. Earlier diagnosis may avoid poor outcomes and PD failure.
